# Clinical impact of high-profile animal-based research reported in the UK national press

**DOI:** 10.1136/bmjos-2019-100039

**Published:** 2020-10-20

**Authors:** Jarrod Bailey, Michael Balls

**Affiliations:** 1 Cruelty Free International, London, UK; 2 University of Nottingham Faculty of Medicine and Health Sciences, Nottingham, UK

**Keywords:** animal research, media, clinical benefit

## Abstract

**Objectives:**

We evaluated animal-based biomedical ‘breakthroughs’ reported in the UK national press in 1995 (25 years prior to the conclusion of this study). Based on evidence of overspeculative reporting of biomedical research in other areas (eg, press releases and scientific papers), we specifically examined animal research in the media, asking, ‘In a given year, what proportion of animal research “breakthroughs”’ published in the UK national press had translated, more than 20 years later, to approved interventions?’

**Methods:**

We searched the Nexis media database (LexisNexis.com) for animal-based biomedical reports in the UK national press. The only restrictions were that the intervention should be specific, such as a named drug, gene, biomedical pathway, to facilitate follow-up, and that there should be claims of some clinical promise.

**Main outcome measures:**

Were any interventions approved for human use? If so, when and by which agency? If not, why, and how far did development proceed? Were any other, directly related interventions approved? Did any of the reports overstate human relevance?

**Results:**

Overspeculation and exaggeration of human relevance was evident in all the articles examined. Of 27 unique published ‘breakthroughs’, only one had clearly resulted in human benefit. Twenty were classified as failures, three were inconclusive and three were partially successful.

**Conclusions:**

The results of animal-based preclinical research studies are commonly overstated in media reports, to prematurely imply often-imminent ‘breakthroughs’ relevant to human medicine.

**Strengths and limitations of this study**

- This study investigates exaggeration in the media of the significance and human relevance of animal research.
- The study focuses on articles in the UK national press in a particular year, and specifically follows up the fate of forecasted ‘breakthroughs’, to see if they had resulted in human benefit >20 years later.
- This study was comprehensive, objective and detailed.
- Significant research was conducted for each media-reported breakthrough, and all its findings have been made available in this report.
- One limitation is that the focus was on one calendar year (1995).
- However, there is no reason to believe that analyses of other years would lead to significantly different conclusions, with regard to the overspeculation and overstatement of potential human benefits from animal-based research in the media.

## Introduction

Animal experiments remain controversial, with issues including the welfare of the animals involved, and the questionable human relevance of animal data.^1–4^ Despite increasing evidence of the latter (see Bailey^5^ for a review), overstatement of the human benefits of animal research is widespread, and occurs throughout the whole research process, from institutional press releases through to reports in the media. For example, Woloshin *et al*^6^ reported that press releases from academic medical centres in the USA ‘often promote research that has uncertain relevance to human health and do not provide key facts or acknowledge important limitations’. Of these, 90% lacked caveats about extrapolating animal/laboratory studies to people, while explicitly making claims about relevance to human health, and 29% exaggerated the importance of the findings they described. Notably, this was much more common for animal studies: 41% were exaggerated in this way, compared with 18% of human studies. Sumner *et al*^7^ examined 462 press releases produced by the UK’s leading 20 (Russell Group) universities, along with the associated scientific papers and print/online news stories, and concluded that 36% ‘contained exaggerated inference to humans from animal research’.

Exaggeration of animal-based findings has also been noted in online and other media. Haneef *et al*^8^ examined the health section of Google News for ‘spin’, and concluded that almost half (48%) of the reports they examined that involved animal studies ‘implied overgeneralization/misleading extrapolation from animals to humans’. The UK’s ‘Leveson Inquiry into the culture, practices and ethics of the press’ concluded that ‘overselling the results of non-human studies as a promised cure potentially confuses readers and might contribute to disillusionment with science’.^9^ The website HealthNewsReview.org published an article in July 2018 about the exaggeration of the applicability and relevance of animal data to humans, based on many of its 6000 posts.^10^ ‘Vigilance’ was advised for both patients and physicians when interpreting health claims that are often exaggerated and/or unfounded, specifically for Parkinson’s disease and other movement disorders, and which included ‘unfulfilled promises of animal models’.^11^

Exaggeration is also evident in scientific publications. Contopoulos-Ioannidis *et al*^12^ examined 101 scientific publications published in top scientific journals (including, but not limited to animal research) and found that basic research rarely impacted clinical practice, even when it was considered ‘highly promising’: 20 years later, only five drugs were licensed for clinical use as a result, and only one was used extensively for the licensed indications. Lindl *et al*^13^ concluded that 17 animal research programmes licensed in Germany in the early 1990s, which promised new therapies, or at least direct clinical impact, had resulted in ‘no clinical relevance’ 17 years later.^14^ Hackam conducted a systematic review to see how often highly cited animal studies from the top seven science journals translated to human success, and concluded that caution should be applied when extrapolating the findings of prominent animal research to the care of human disease.^15^ Of 76 qualifying animal studies, 28 had positive outcomes in human trials; but only 8 led to therapies approved for clinical use.^15^

In summary, in the past approximately 15 years, various efforts have been made to assess the outcomes and human benefits of scientific breakthroughs, and how accurately and speculatively these were reported. Overstatement, overspeculation and exaggeration were highly prevalent. We sought to explore these issues further, and uniquely, by examining reports of animal-based biomedical ‘breakthroughs’ in UK national newspapers in 1995, 25 years prior to the conclusion of this study in 2020. Our aim was to determine whether any of these biomedical ‘breakthroughs’ had resulted in clinical benefit, and to what degree their clinical impact had been exaggerated. This period provides ample time for the apparent ‘breakthroughs’ to be developed, tested and ultimately translated into clinical benefit, and is a similar time span to that used in a comparable study.^12^ We also wanted to investigate whether, if any breakthrough had been realised, it depended on the animal studies, and if no direct breakthrough had resulted, any related breakthroughs could be linked to the reported animal research.

## Methods

The ‘Nexis’ database is an archive of more than 40 000 information sources of various types, including news content, provided by the international company, LexisNexis (lexisnexis.com). Media sources were selected to include ‘UK national newspapers’, in the calendar year 1995. The search strategy involved selecting the ‘Medical research’ index term, then adding the following animal terms to identify news items based on animal research: ‘animal OR mouse OR mice OR rodent OR rat OR dog OR cat OR monkey OR primate OR guinea pig OR rabbit’. Articles that did not describe a clear, direct clinical promise, or that described a non-clinical application (eg, agricultural or veterinary), or that described *only* mechanisms of action, pathophysiology or diagnosis, or in which the intervention was not of a specific named procedure or compound, or was not speculated to be associated with a specific gene/molecule/pathway, were excluded. For each report, the associated academic publication(s) were obtained, where available, and as much of the following data that were available were extracted: title, news media source, publishing journal, date of publication, author name(s), PubMed ID and links, animal species and numbers used, intervention, preventive/therapeutic in nature, expected clinical benefit and years to expected benefit, relevant text and summary of findings, disease in question, institution, funding body, harms to animals, any salient quotes from authors, any obvious related material, etc.

To investigate whether clinical benefit transpired within 20-plus years, the following websites and sources were consulted: PubMed, the European Medicines Agency, the UK Medicines and Healthcare Regulatory Agency, the US Food and Drug Administration (FDA), clinicaltrials.gov, Medscape.com, the National Institute for Health and Care Excellence, the British National Formulary, Centers for Disease Control and Prevention, the WHO and the US National Library of Medicine’s TOXNET. Data obtained from thorough searches of these sources were collated, and used to determine the outcome of each ‘breakthrough’ with regard to any further studies that were conducted; whether these were human, animal or both; if clinical trials were conducted, and what the results of these were with respect to efficacy and adverse drug reactions; if the drug/intervention reached the market, and if so, if it had been relabelled or recalled. Based on the above, a decision was made, in consultation with colleagues, about whether the 1995 media report had been accurate in reporting the research as a ‘breakthrough’. For clarity: if the intervention in question had not been approved at the time of writing, >20 years after the media report, it was classified as ‘failed’. Some were classified as a ‘partial success’, if, for example, use was restricted clinically and/or geographically; any use was specific to particular, rather than general, circumstances (ie, a narrower use than had been claimed); an approved therapy was of questionable efficacy; evidence from other, non-animal research data (including human data) suggested the animal data were not crucial to the ‘breakthrough’; there was an indirect relation between the ‘breakthrough’ and the successful intervention; there was questionable clinical relevance of the animal data and so on.

## Results

The initial search produced 229 articles, and the removal of 16 duplicates left 213 for consideration according to the selection criteria. Forty individual articles (reporting 42 animal-based scientific ‘breakthroughs’) met these inclusion criteria. Some of these breakthroughs were (not surprisingly) reported in more than one article: grouping duplicates together resulted in 27 unique ‘breakthroughs’. These involved a variety of diseases, conditions and biomedical areas, including HIV/AIDS, malaria, allergies, Alzheimer’s disease (AD), multiple sclerosis, deafness, cancers, obesity, pain, organ transplantation, ageing and others. Each newspaper article reporting breakthroughs in these areas contained speculative claims, from scientists undertaking the research, as well as from the reporters and others involved: in other words, overstatement of the potential relevance of the breakthroughs came from scientists as well as the journalists involved. These are some of the more speculative examples, to illustrate what was found. With regard to an anti-allergy vaccination, ‘This is potentially one of the biggest-selling drugs ever…the company estimates that the vaccine will be available in the UK in five years’. In cancer gene therapy, ‘It is hoped that by giving the correct version of the gene to lung cancer patients, normal apoptosis will resume and the tumours will shrink. Experiments carried out on animals are encouraging’. With regard to obesity treatments, ‘There seems no doubt that this new technology will appear: the only question is when…within a few years a fat reducing injection could make liposuction a thing of the past…the fact that it has been seen to work in several animal species shows that it is very likely to work in humans, too’, and ‘This is a major breakthrough in obesity research…We have every reason to believe this could become a treatment for obesity in humans’. In organ transplantation, ‘The first organ transplants from pigs to humans are expected to begin next year in a move that could signal an end to the global shortage of human donors…rejection problems involved in xenotransplantation are being solved’, and in ageing research, “Researchers have discovered a natural hormone produced by the body that could delay the effects of ageing…the hormone could help to defer such characteristic problems of old age as wrinkles, muscle fatigue, rheumatism, bone fragility, memory loss and some cancers…the results so far in animals had been “spectacular”’.

**Figure 1:**
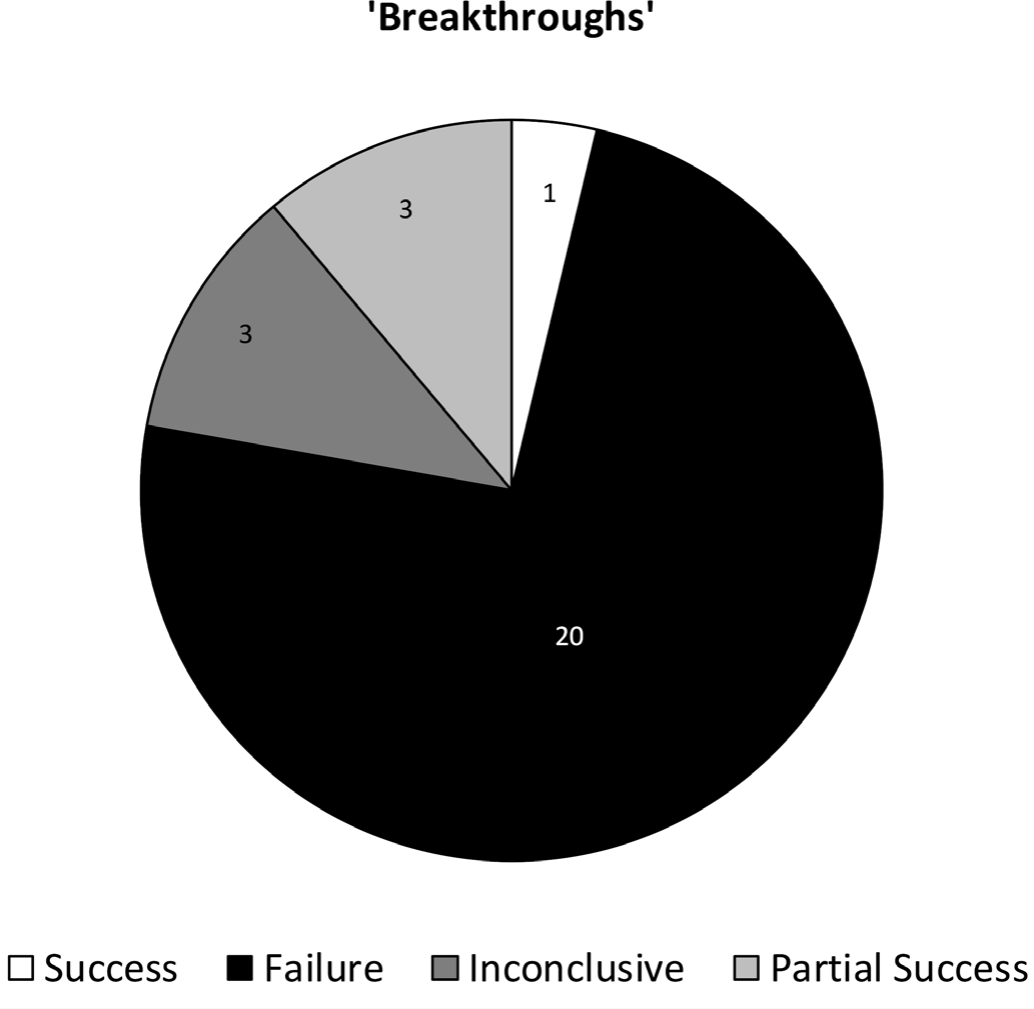
‘Breakthroughs’: proportion of successes, failures, partial successes and inconclusive outcomes. Only one of the 27 unique animal-based ‘breakthroughs’ could be considered successful (#11, the Norian Skeletal Repair System). Twenty of the 27 were outright failures, with no direct human clinical benefit. Of the remaining six: three were inconclusive, and three ‘partially successful, with caveats’. These results indicate a failure rate of 26 out of 27, and an outright success rate of only 1 out of 27.

Table 1 shows a brief summary of each ‘breakthrough’, with multiple reports of the same ‘breakthrough’ grouped together. The information given includes a concise description of each discovery and its clinical promise, the reporting media article, any further research and development and an evaluation of the final outcome. Three examples of these detailed discussions—one for each case of success, partial success/inconclusive and failure—are provided below, to illustrate the thorough nature of our research. Just one of the 27 unique ‘breakthroughs’ reported in the 40 articles in the UK national press that met the inclusion criteria, was classified as an outright success. Twenty were classified as outright failures, with no direct clinical benefit. Of the remaining six, three were classified as inconclusive (either because clinical trials were ongoing, or because the evidence was mixed), and three were classified as a ‘partial success’ (see ‘Methods’ section for details). The overall results are summarised in figure 1.

**Table 1:** ‘Breakthroughs’ listed according to results from LexisNexis database

No(s).	Intervention	News item(s)	Further research/Status	Overall outcome
1	Antisense-DNA therapy for leishmaniasis	Once Bitten. *The Times*, 10 January 1995, Nigel Hawkes	Further animal studies conducted, but no human studies found.	*Failed*. There is no antisense therapy for leishmaniasis.
2 22	Baboon bone-marrow transplant for HIV/AIDS	Lifeline from a baboon; AIDS victim pins hopes on monkey bone-marrow. *Daily Mail*, 18 December 1995 AIDS Patient to be Given Baboon Marrow. *The Times*, 11 July 1995, Giles Whittell	No further animal or clinical studies found.	*Failed*. Procedure unsuccessful. Not used clinically.
3	Malaria vaccine produced in edible plants	A green way to health. *The Times*, 23 January 1995, Nigel Hawkes	No further animal or clinical studies found.	*Failed*. No plant-based malaria vaccine exists.
4	Anti-allergy vaccine	Ultimate allergy shot; Innovation: British company boasts of a vaccine with huge potential. *The Independent,* 29 January 1995, Nuala Moran	Clinical studies: negative human efficacy outcome (adverse reaction outcomes uncertain).	*Failed*. No general anti-allergy vaccine approved.
5	Attenuated-HIV live-virus vaccine	Vaccine hope for AIDS. *The Times*, 30 January 1995, Nigel Hawkes	No further animal or clinical studies found.	*Failed*. No attenuated live HIV/AIDS vaccine approved.
6	New improved mouse model of Alzheimer’s Disease, for better testing of new therapies	Alzheimer's mouse may hold clue to cure. *The Times*, 9 February 1995, Nigel Hawkes	Clinical studies: negative human efficacy outcome; adverse reactions	*Failed*. No successful AD treatment from this mouse model exists.
7	New Anti-Depressant Drug is Also Treatment for Multiple Sclerosis (MS)	Drug Hope for MS Sufferers. *The Times*, 2 March 1995, Nigel Hawkes	Clinical studies: positive human efficacy outcome; adverse reactions	*Failed*. Rolipram not in clinical use due to serious adverse effects.
8	Identification of gene causing some childhood deafness and blindness brings hope of cure	Mouse Gene Points Way to Site of Child Hearing and Sight Defect. *The Guardian*, 3 March 1995, Tim Radford	Clinical studies: awaiting results	*Failed*. No therapy associated with this gene/pathway exists.
9	Inhibitors of *ras* genes could be a cure for many cancers	Stopping cancer in its tracks. *The Times*, 21 March 1995, Nigel Hawkes	Clinical studies: negative human efficacy outcome; no adverse reactions	*Failed*. No *ras* inhibitor approved as anticancer treatment.
10	New drug for Alzheimer’s Disease (GTS-21)	Database—Fags for the memory. *The Observer*, 9 April 1995, Nina Hall	Clinical studies: negative human efficacy outcome; no adverse reactions	*Failed*. GTS-21 is not used to treat dementia.
11	Mending broken bones with injectable ‘Skeletal Repair System’	Cast Away Your Plaster Cast. *The Times*, 25 April 1995, Nigel Hawkes	Marketed: some adverse reactions.	*Success (with caveats)*. Used for specific purposes, with caution.
12	Curing cancer by *p53* gene therapy	Why Our Cells Must Perish. *The Times*, 1 May 1995, Nigel Hawkes	Clinical studies: negative human efficacy outcome; no adverse reactions	*Failed*. Only approved *p53*-based therapy is in China.
13 15	Injection to treat obesity/‘end the need to slim or diet’	Anti-obesity jab may end need to diet or exercise. *The Times*, 16 May 1995, Nick Nutall Can this injection get rid of your fat forever. *Daily Mail*, 23 May 1995, Jane Alexander	Further animal studies conducted, but no human studies found.	*Failed*. No antibody-based obesity treatment approved.
14	Cure for cancer (solid tumours)	Trials begin on pill that could control cancer. *The Independent*, 19 May 1995, Celia Hall	Clinical studies: negative human efficacy outcome; adverse reactions	*Failed*. Marimastat not in use.
16a	Cannabis to treat chronic pain	Try a little flower power; Long dismissed as unscientific, plants are making a pharmaceutical comeback, says Roger Dobson. *The Independent*, 23 May 1995, Roger Dobson	Clinical studies: inconclusive human efficacy outcomes; no adverse reactions	*Inconclusive*. Mixed evidence.
16b 19 30	Treatment for Alzheimer’s Disease with Extract of Daffodils (Galanthamine)	Try a little flower power; Long dismissed as unscientific, plants are making a pharmaceutical comeback, says Roger Dobson. *The Independent*, 23 May 1995, Roger Dobson The Brain’s Messenger. *The Times*, 12 June 1995, Nigel Hawkes Drug Hopes Rest on a Host of Daffodils; A Bulb Extract May Alleviate Alzheimer's—and Boost East Anglia Growers. *The Independent*, 3 September 1995, Michael Leapman	Controversy surrounding human efficacy and safety.	*Partial success*. Approved in some countries, but questions remain regarding efficacy and safety.
18	Sugar on the tongue can ease pain (in place of pain-relieving drugs) in young babies	Sugar ‘Eases Pain in babies’. *The Independent*, 9 June 1995, Liz Hunt	Mixed outcomes/evidence base and caveats	*Partial success*. Evidence supports use for some procedures, but other interventions are as (or more) effective.
20	Discovery of genetic mutations that result in life extension for nematode worms, touted as a lead in anti-ageing interventions for humans	Clue to Long Life Unearthed. *Daily Mail*, 19 June 1995	Further animal studies conducted, but no human studies found.	*Failed*. No antiageing therapy based on this gene approved.
21	New, improved vaccines based on DNA (eg, for influenza immunisations)	New Vaccine Made to Order. *The Times*, 19 June 1995, Dr John Turney	Clinical studies: negative human efficacy outcome; no adverse reactions	*Failed*. No human DNA vaccines approved.
23a 25 26 27 29 34	Daily injections of the hormone leptin could be a cure for obesity	Could These Mice Help Women to Look Like This; Forget That Diet: A Daily Jab May Soon Be Enough. *Daily Mail*, 28 July 1995, George Gordon Drug Firms ‘Hyping Research on Obesity’; Steve Connor on the Row Over Tests for an Injectable ‘Cure’ for Fatness. *The Independent*, 28 July 1995, Steve Connor ‘Miracle’ Cure for Fatness has Slim Chance of Success. *The Independent*, 30 July 1995, Steve Connor Diet Hormone Makes Mice Thin, but Not Humans; Research Suggests Hopes of Obesity Cure Were Premature. *The Independent*, 1 August 1995, Steve Connor The Mouse That Could Make Us Thin. *The Times*, 29 August 1995, Kate Muir How to Lose Weight Without Trying. *The Independent*, 19 September 1995, Sarah Edghill	Clinical studies: negative human efficacy outcome; no adverse reactions	*Failed*. Leptin treatment for obesity not approved.
28	Research on dieting people and genetically-modified mice suggests new target for weight-loss drug development	Brain Chemical May Hold Secrets of Why Diets Fail to Work. *The Times*, 17 August 1995, Nigel Hawkes	One related therapy approved in the USA, with questionable efficacy. Further research ongoing.	*Partial success*.
23b 31 32 33	Genetically-modified pig organs will successfully address the shortage of organs for human transplant	Could these mice help women to look like this; forget that diet: a daily jab may soon be enough. *Daily Mail*, 28 July 1995, George Gordon (Brief mention of animal organ transplants in article focusing on obesity jabs) Pig Hearts Could End Fatal Lack of Transplant Organs. *The Times*, 14 September 1995, Jeremy Laurance The moral implications of animal transplants will disturb many. But an eminent Cambridge don says we should rejoice; 'Pigs will be tailored for each of us so we have organs for emergencies. Godparents may give children their own pigs, bred on a scientific farm'. *Daily Mail*, 14 September 1995, Dr Terence Kealy Pioneer spurned by Britain. *The Independent*, 17 September 1995, Charles Arthur	Further animal studies conducted, but no human studies found.	*Failed*. Xenotransplantation of non-human organs to humans does not take place.
35	Extending tamoxifen prescribing for breast cancer patients could increase survival; but would liver cancer be an issue, as suggested by rodent studies?	Drug Test on Women with Breast cancer. *The Guardian*, 13 October 1995, Chris Mihill	Clinical studies: negative human efficacy outcome; no adverse reactions	*Failed*. Evidence did not affect use of tamoxifen.
36	Mouse experiments suggested the natural hormone melatonin could reverse the ageing process, and promote rejuvenation and extend healthspan, also in humans	Melatonin; It Has Been Hailed as a Wonder Drug that can Cure Insomnia and Reverse Ageing. So Why is it Being Banned? *Daily Mail*, 17 October 1995, Jemima Lewis	Clinical studies: negative human efficacy outcome; no adverse reactions	*Failed*. Melatonin is not approved for therapeutic purposes related to ageing.
37 38 39 40	Growing tissues in the laboratory for transplantation: the famous/infamous ‘mouse with an ear on its back’ experiment	Is This a Breakthrough to Benefit Mankind … or Science Running Amok? *Daily Mail*, 24 October 1995, Tracey Harrison Of Mice, Men and Wacky Medicine; Off-the-Shelf Skin Cloned from the Human Body? *The Guardian*, 28 October 1995, Tim Radford Genetic Engineering: Tinkering with the Destinies of Mice and Men. *The Observer*, 29 October 1995, Judy Jones S & N's $ 1bn Science Fiction Adventure. *Daily Mail*, 30 October 1995, Michael Walters	Clinical studies: mixed outcomes, depending on specific tissue type/application.	*Partial success*. Main promises not realised; research ongoing.
41	Newly-discovered antigens impact success of organ transplants between sexes: new drugs targeting them will help	Gender Transplants. *The Sunday Times*, 9 November 1995	Further animal studies conducted, but no human studies found.	*Failed*. No drugs related to this finding are in use for organ transplantation.
44	Naturally-occurring hormone dehydroepiandrosterone (DHEA) promises to reverse the adverse effects of ageing	Natural Hormone May Soften the Blows of Age. *The Times*, 9 January 1995, Nigel Hawkes	Mixed evidence—still unknown.	*Failed*. DHEA not approved for ageing therapy.

Multiple reports of the same ‘breakthrough’ are grouped together. Brief summaries are shown. Forty media articles reported 42 animal-based breakthroughs: due to multiple reports, the number of unique breakthroughs was 27. For each of them, the table shows: the article identification number; brief description of the intervention/discovery; article title, publishing newspaper, date and author; clinical promise of intervention/discovery; further research and final evaluation of the outcome.

### Examples of detailed discussions of each ‘breakthrough’

These examples—one from each of the main classifications (success, partial success and failure)—are included here, to illustrate the detailed and comprehensive investigation and follow-up conducted by the authors, to ensure that the classifications are as accurate as possible. They were selected subjectively by the authors, as being particularly illustrative and of interest.

### Success

11. Mending broken bones with injectable ‘Skeletal Repair System’—Succeeded (with caveats)

Cast Away Your Plaster Cast—*The Times*, April 25 1995

*The Times* reported the development of an injectable paste that can be introduced—‘like toothpaste’—into broken or fractured bones, or bones affected by osteoporosis.

This bone substitute—Skeletal Repair System (SRS)—was invented by Norian Corporation (USA). It was not the first bone substitute intended to replace metal implants, but was claimed to be a better match for real bone than anything that had gone before it.

The report cited a paper in *Science*, stating ‘Experiments with animals have given good results, and the first tests on human patients…have produced good repairs of broken wrists’.^16^ Rabbits and dogs were involved: bone sections were removed from the ulnas of 12 rabbits, and cement injected. The rabbits were X-rayed, and killed ‘at 12 weeks’ for tissue examination. Human investigations involved repairing the fractured distal radius of a woman aged 49 years, for whom X-rays showed ‘stabilisation’ and ‘maintenance of correct position’ following injection of SRS.

A 2003 paper discussing the background to SRS^17^ cited six human investigations, from 1966 to 1995, and reported human clinical investigations of SRS during the 5 years after *The Times* report, between 1996 and 2000,^18–20^ as well as the authors’ own clinical research. They did, however—in common with some previous human data—report that ‘The risk of extrusion of the SRS cement into undesirable locations has been a substantial concern’, leading to a higher complication rate.

More recently (2012–2013): Ozer and Chung^21^ cited the papers above,^17–19^ as well as uncontrolled case series from 2003^22^ and 2007^23^ that showed SRS was safe and supportive. Dorozhkin’s review^24^ reported problems with SRS use, including a high rate of infectious complications, which led some to discontinue SRS for some specific uses.^25^ SRS had shown high infection rates in other human studies,^26–28^ as well as cement fragmentation^27^ and wound dehiscence.^28^

A 2010 meta-analysis noted bone cements were first introduced in ceramic form in 1992^29^—3 years prior to *The Times* article and the associated paper.^16^ The ‘paste form’ became available in the ‘early 1990s’, again before these publications. It also noted caveats regarding long-term results, and complication rates of 13%–31%.^30 31^ Overall, reported complication rates were up to 62%, and were often serious and extended for many years after surgery.^29^

Norian was bought by Synthes in 1999, when SRS had been approved for use in the arm, and another version, Craniofacial Repair System (CRS), for use in the skull. Ten years later, Synthes was accused of ‘running illegal clinical trials—essentially, experimenting on humans’. They had mixed SRS with barium sulphate, in a new formulation known as XR, to facilitate visualisation on X-rays. Although XR had been approved by the US FDA in 2002, it had expressly *not* been approved for use in certain spinal surgeries, such as the treatment of vertebral compression fractures—a common consequence of osteoporosis. This was due to concerns over Norian cement leaking into blood vessels—numerous in the spine—which, it was known, could cause blood clotting, with severe or lethal consequences.

In 2009, Norian wanted to begin using a new formulation—XR—in spinal surgeries, as they considered it would be lucrative, but the US FDA ordered them to conduct lengthy and expensive clinical trials. Instead, they persuaded ‘a few sites’ to perform 60–80 human procedures and publish the results—quicker and cheaper, but at least five people died. This had taken place despite data highlighting its risk: small amounts of XR had caused human blood to form clots in test tubes, suggesting blood vessels in patients’ hearts or lungs could also be blocked. In addition, the injection of XR into a pig’s vein had caused clots in its lungs that killed it within seconds. The company pleaded guilty to dozens of felonies and misdemeanours, was fined US$23 million, and four of its executives were imprisoned.

It must be concluded, therefore, that even though SRS was approved for human use, the animal data did not predict many of the major complications of SRS use that were revealed by research with humans. While SRS remains in use, it is used with caution and only for particular purposes, and certain caveats must be borne in mind—all as a result of human studies.

### Partial success

16b, 19 and 30. Treatment for Alzheimer’s Disease with Extract of Daffodils (Galanthamine)—Partly successful, with caveats

Try a little flower power; Long dismissed as unscientific, plants are making a pharmaceutical comeback, says Roger Dobson—*The Independent*, May 23 1995

The Brain’s Messenger—*The Times*, June 12 1995

Drug Hopes Rest on a Host of Daffodils; A Bulb Extract May Alleviate Alzheimer's - and Boost East Anglia Growers—*The Independent*, September 3 1995

An extract of daffodil and snowdrop bulbs was proposed to slow the progress of AD. Brain-damaged rats, deficient in the neurotransmitter acetylcholine (ACh), showed a slower rate of learning to navigate through water mazes. This is consistent with poorer memory in patients with AD, whose brains show a deficiency in ACh, although there remains controversy over whether this is cause or effect. Rats genetically modified (GM) with cells that replaced the lost ACh could navigate mazes better than those who had not: ACh replacement appeared to restore memory function and learning deficits. It was hoped that this would lead to drugs designed to halt the decline of ACh associated with AD. However, a drug to do this was already in clinical trials (galanthamine, extracted from daffodil bulbs), and another—Tacrine—had already been licensed in some countries. A subsequent article in *The Independent* briefly discussed the progression of galanthamine into clinical trials, which had reached phase III, involving 560 patients across Europe.

The paper described how rats had their brains damaged via direct injections of ibotenic acid, causing ‘permanently and selectively damaged learning and memory’.^32^ The ‘ACh-replaced’ rats had GM cells grafted/infused into their brains, and 4 weeks later were killed for analysis by decapitation. The need for this harmful study is open to question, given the weight of evidence implicating the cholinergic system in memory and learning. The authors themselves cited previous research that did this, including both rat experiments and human research.^33–35^ The stated value of their study was that it ‘had not been *proved* that regional ACh is causally required for learning and memory’. Furthermore, drugs to address this issue were already in use and in clinical development, so in no way could have depended on these particular animal experiments. This was tacitly acknowledged by the authors.

It is worth examining the path of galanthamine to clinical trials, particularly for the contribution (or lack of) of animal experiments. It was discovered accidentally in the early 1950s, and used for various purposes since then, including nerve pain, polio and in anaesthesiology.^36^ It has been extensively investigated in humans, showing memory enhancement properties, although with some adverse effects; and derivatives have been sought and tested to overcome these effects.

There was extensive, promising, human research preceding the 1995 ‘breakthrough’, in both patients with AD and healthy volunteers.^37–40^ A 2004 review showed galanthamine had been used for many years in Eastern Europe, prior to its preclinical testing in Western Europe in the 1980s.^41^ In the 1950s, it was used to ease nerve pain, and to treat polio; preclinical experiments continued throughout the 1980s, and some salient research involved ex vivo muscles from frogs, leeches and rabbits, rather than experiments involving live animals, to investigate its inhibitory properties for acetylcholinesterase.

Clinical development progressed throughout the 1990s; and it was first licensed for AD treatment in 2000 in Iceland, Ireland, Sweden and the UK, followed by the USA and other countries in the early 2000s. There have been significant issues, however. While some trials showed it to be well tolerated and to improve cognitive function in patients with AD,^42 43^ two large trials did not show a significant difference from the effects of the placebo, with regard to rate of progression of AD.^44^ One 2018 review noted that clinical trials were ‘still ongoing’.^45^ Other major caveats, including with other cholinesterase inhibitors, donepezil and rivastigmine, included that they are effective for a maximum of about 3 years, and also that they treated only AD symptoms, not the disease itself.^46^ Other caveats are still being reported: galanthamine treatment is ‘still saddled with numerous side effects’.^47^

In summary, there was substantial, significant weight of evidence of the role and ACh in AD prior to the 1995 rat experiments; much of this was human specific and much of this was acknowledged by the authors themselves. Drugs targeting this pathway were already in clinical development, and so it cannot be claimed that galanthamine development depended on animal research—and certainly not on this particular research—due to the extensive human data relating to it, which go back hundreds of years, and which include detailed pharmacodynamic and pharmacokinetic data preceding 1995. Human trials are still ongoing, and it is they that will clarify issues regarding safety and efficacy.

### Failure

23b, 31, 32, 33. Genetically modified pig organs will successfully address the shortage of organs for human transplant—Failed

Could these mice help women to look like this; forget that diet: a daily jab may soon be enough—*Daily Mail*, July 28 1995 (Brief mention of animal organ transplants in article focusing on obesity jabs)

Pig Hearts Could End Fatal Lack of Transplant Organs—*The Times*, September 14 1995

The moral implications of animal transplants will disturb many. But an eminent Cambridge don says we should rejoice; ‘Pigs will be tailored for each of us so we have organs for emergencies. Godparents may give children their own pigs, bred on a scientific farm’—*Daily Mail*, September 14 1995

Pioneer spurned by Britain—*The Independent*, September 17 1995

In the main article, *The Times* reported the expected commencement of clinical trials of pig-to-human organ transplants—xenotransplantation (XTP).

These trials would be based on experiments in which hearts from GM pigs survived for up to 60 days in monkeys who had received transplants, supported by immunosuppressive drugs to help prevent rejection—a perennial issue with organ transplantation, but particularly with transplantation between different species. The article was optimistic: the director of the company directing the experiments, Imutran, claimed ‘a big hurdle in the development of transplants between species known as xenotransplantation had been overcome’; ‘the rejection problems involved in xenotransplantation are being solved’ and that they had ‘found a way to trick the immune system of a primate into accepting a pig organ’, while the director of transplant services at Papworth Hospital stated, “If progress continues the way it is, we intend to start human clinical trials in 1996”', and that it would be at least 5 years before animal transplants were generally available. At the same time, the article noted the urgent need for organs for transplantation, stating that ‘The first organ transplants from pigs to humans are expected to begin next year in a move that could signal an end to the global shortage of human donors’, and ‘If successful, the technique could open up the prospect of animal transplants to thousands more patients who are denied treatment because of a shortage of human organs’.

A paper in *Nature* carried an associated report.^48^ The medical director of Imutran (David White) was quoted again, reporting that 10 monkeys with pig hearts had survived an average of 40 days, with two surviving for >60 days. The basis for this improved survival was that the new GM pigs, providing donor hearts for the monkeys, had been genetically engineered in an attempt to overcome hyperacute rejection. This is the almost immediate rejection of an organ following transplantation, which can occur within minutes. There are other types of rejection that may occur subsequently: acute/acute vascular rejection, which can take several days, and chronic rejection, which can take years. However, White was dismissive of concerns about such levels of confidence being premature, and about assertions that much more understanding of the mechanisms of transplant rejection was needed, stating, “As far as we can see, the other hurdles have not raised their head of (*sic*) the timeframe of our experiments”.

Building on this: GM pigs were created, with genes for two regulators of complement activity.^49^ The following year did not see any human trials commence, however, and transplantations were not taking place within 5 years, as promised. In fact, while research has progressed, the intervening quarter of a century has revealed numerous and unforeseen challenges, and human trials still seem distant. First, Imutran and associated companies closed down. Other companies and researchers pressed ahead, and failed to deliver on earlier promises, with failure after failure. Major immunological barriers have manifested, with recent publications confirming that organ rejection is still a major issue. For example, although one author considered survival times of 90 days in their research ‘impressive’,^50^ orthotopic heart transplants from pigs to baboons were associated with a maximum survival of 195 days, though this particular animal had to be killed due to signs of heart and liver dysfunction.^51^ The International Society of Heart Lung Transplantation suggested that clinical trials of heart XTP should be considered when pig hearts could be transplanted into non-human primates (NHPs), with predefined immunosuppression, with ‘60% survival at 3 months and a minimum of 10 animals surviving for this period’,^52 53^ but this has still not happened, even though some claim that this goal may be attainable.^53^ Furthermore, most experiments have involved heterotopic, rather than orthotopic, transplants, in which the transplanted organ is placed away from its normal site in the abdomen, which is non-life supporting; orthotopic transplantation, where the organ is placed in its usual site, in order to support life, will be required by the regulatory authorities.^54^

Additionally, issues with the transfer of pathogenic microorganisms from the donor pigs to organ recipients continue, despite significant efforts to combat them. Other variables affecting survival include immune suppression, donor genetics, recipient species (ie, humans will probably react differently to NHPs), viral status, the level of pre-existing antipig antibody, prophylactic antiviral and antibacterial therapy and postoperative care.^53^ A 2018 review noted that, while survival had increased over the years (decades) ‘from days to months’, ‘additional barriers due to antigenic and physiologic differences in cross-species transplantation continue to remain a challenge’.^55^

Ongoing work towards human trials centres around increasing ‘tolerance’ via multiple genetic modifications of pigs, targeting the many (and increasing) antigens involved in organ rejection. The current level of immunosuppression required to prolong survival post-XTP is still unacceptably high, and so even greater genetic modification of pig donors is necessary.^56^ This is already high: multitransgenic pig kidneys containing five modified genes have been tested in baboons: one combination allowed survival of 6 months or more, while another still resulted in serious problems, leading to the conclusion that ‘the exact responsible genes have yet to be identified’.^55^

It therefore must be asked; how much genetic modification might permit an adequate level of survival? And, even if it were possible, could it ever be enough? This may be illustrated by the identification of another crucial antigen involved in rejection, B4GALNT2.^57^ One initial ‘success’ of GM pigs was the knockout of the Gal gene—but while this helped resolve Gal-mediated rejection, it ‘did not eliminate antibody-mediated rejection and instead highlighted the importance of antibody directed to non-Gal pig antigens’. Many other antigens have been implicated, and others remain to be discovered. Reviews from 2017 to 18 detail the complexity of XTP organ rejection, and the numerous genetic modifications created in attempts to overcome it: 26–30 different modifications in pigs, involving genes associated with Gal, complement regulation, cellular immune response, anticoagulation, anti-inflammatory, anti-apoptotic and other pathways, and noted that other, new antigens were being discovered that may require further genetic modifications.^58 59^

Within a few days of the article in *The Times*, two other articles were printed. One in the *Daily Mail* was an overly speculative positive spin on results from Imutran, in which quotes illustrated comprehensively the issue of exaggeration and embellishment. The other, in *The Independent*, focused on why British venture capitalists failed to back Imutran, and was also replete with positive spin. In it, a major financial backer appreciated that ‘These things take between 6 and 10 years to mature’—but a quarter of a century later, we are still waiting.

## Discussion

Our analysis demonstrates that the relevance and impact of animal-based findings to human health are frequently overstated in the UK national press—findings that are consistent with similar reports of exaggeration in institutional press releases, online news, etc,^6–11^ and which are not limited to English-speaking researchers and English-language media.^60^ Recent publications have accepted and addressed this, by recommending increased accuracy and detail in academic press releases and subsequent media articles, which should include more explicit caveats and more cautious language—and that this can be done without harming public interest in the news.^61–63^ There is some evidence of this being implemented, in that more media articles reporting results from animal experiments now do so explicitly, instead of leaving the reader wondering if the research was done in humans or animals.^61 62^

Similar observations have also been made for publications in scientific journals (see ‘Introduction’).^12–15 64 65^ In addition, for instance, Kilkenny *et al* highlighted serious omissions in the way that research using animals is reported (eg, in experimental design, description and statistical analysis), recommending that authors should explicitly comment on limitations of animal data and their relevance to humans.^66^ ter Riet *et al* examined publication bias in animal research, revealing that ‘negative’ animal results were rarely published, leading to a publication bias that ‘will impede the performance of valid literature syntheses’, as this invariably must lead to an inflated view of the success of animal studies.^67^

We found that only one of 27 original breakthroughs reported in the UK national press in 1995 had led unambiguously to clinical benefit >20 years later. This result carries more weight than it might have, because we made attempts, where no direct breakthrough was evident, to determine whether any related breakthroughs had resulted from the animal experiments, and if so, whether the animal research had been essential. Even if any of the six ‘breakthroughs’ currently classified as ‘inconclusive’ or a ‘partial success’ are reclassified as a success in the future, the degree of successful translation—and therefore exaggeration—are still disappointing, especially given the high ethical and economic costs of animal research.

Classifying some of the ‘breakthroughs’, for example, as ‘partial successes’ or as ‘failures’, was not straightforward. Some areas of research had taken a related, but different, direction; some results effectively duplicated, or at least were strongly underpinned by, previous human research and/or animal research; some interventions had complex and changing nomenclatures; some were broad in nature (eg, the identification of a gene, rather than the testing of a specific intervention); some had gone on to clinical trials with unpublished results and so on.

In terms of limitations, our study focused on one calendar year. While there may be some degree of variability from year to year with regard to the areas of animal research and the degree of the translation to human benefit reported in the media, we employed broad selection criteria, reflected in a wide variety of research topics, and are therefore confident that these particular aspects of our findings can be generalised. We accept that our analyses could not include animal research that was not expressly reported as animal research, that is, that was conveyed as if it already applied to humans. Our search strategy could not identify such reports, but it was not the aim of our work to do this; however, our goal was to examine research clearly done in animals, as reported in the UK national media.

Our findings, along with the observations of the other authors cited, should encourage media reports of animal research ‘breakthroughs’ that forecast benefits to human health to be viewed with caution. This is of crucial importance, as widespread and high-profile dissemination of exaggerated and overspeculative claims will lead to general overconfidence among the public—as well as the research community—of animal research as an approach, and an overly optimistic assumption of eventual clinical benefits.^68^ There are also implications for the policies of governments, regulators and funders of biomedical research, institutional and personal advocates and practitioners of animal research and other stakeholders. Ideally, the culture ingrained in the scientific community—which is to some degree understandable, given the competition for funds and need to justify research—of embellishment of research results in all communications, from grant applications and institutional press releases, through to papers in scientific journals and associated media reports, must be addressed, perhaps with policy decisions. This is especially necessary for research on animals, with its associated welfare implications, which can be severe.
